# Is Laparoendoscopic Single-Site Adrenalectomy a Feasible Alternative in Treating Aldosterone-Producing Adenoma?

**DOI:** 10.1155/2016/6894381

**Published:** 2016-11-16

**Authors:** Che-Hsiung Wu, Leay-Kiaw Er, Ya-Hui Hu, Chia Da Lin, Shih-Chieh Chueh, Yao-Chou Tsai

**Affiliations:** ^1^Division of Nephrology, Taipei Tzuchi Hospital, The Buddhist Tzu Chi Medical Foundation, Taipei, Taiwan; ^2^Division of Endocrine and Metabolism, Taipei Tzuchi Hospital, The Buddhist Tzu Chi Medical Foundation, Taipei, Taiwan; ^3^Department of Surgery, Taipei Tzuchi Hospital, The Buddhist Tzu Chi Medical Foundation, Taipei, Taiwan; ^4^Department of Urology, Tzu Chi University, Medical College, Hualien, Taiwan; ^5^Glickman Urologic and Kidney Institute, Cleveland Clinic, Cleveland, OH, USA; ^6^Cleveland Clinic Lerner College of Medicine, Case Western Reserve University, Cleveland, OH, USA

## Abstract

*Objective*. To compare laparoendoscopic single-site (LESS) and conventional multiport adrenalectomy in patients with aldosterone-producing adenoma (APA).* Material and Methods*. We retrospectively reviewed patients who had been clinically confirmed with unilateral APA and who underwent LESS or multiport adrenalectomy between 2009 and 2014. Perioperative data were obtained for all patients. Blood pressure and the levels of serum aldosterone, renin, and potassium were checked periodically.* Results*. We identified 45 APA patients in the LESS group and 71 in the multiport group. The baseline characteristics were matched between two groups. All adrenalectomies were completed successfully, except one with laparoscopic conversion in the single-port group and one open conversion in the multiport group. After a mean follow-up around one year, there were no significant group differences in the improvement of hypertension, number of types of medication taken, and cure of hypokalemia after operation.* Conclusions*. Our study confirm that LESS adrenalectomy achieved similar clinical and functional outcomes as conventional multiport adrenalectomy for management of unilateral APA.

## 1. Introduction

Primary aldosteronism (PA) is one of the few surgically curable causes of hypertension. Most PA is caused by an aldosterone-producing adenoma (APA) in the unilateral or bilateral adrenal gland. According to a screening study in Japan, PA is found in 3.3–10% of hypertensive patients and is the most common cause of secondary hypertension [1–3]. Thus far, unilateral adrenalectomy is still the treatment of choice in patients with unilateral APA.

Laparoscopic adrenalectomy has become the recommended procedure for benign adrenal mass due to reduced postoperative pain and shorter convalescence when compared with open surgery adrenalectomy [4, 5]. A recent revolution in surgery, the endoscopic single-port surgery or laparoscopic single-site surgery (LESS), has been implemented in various surgical indications including adrenalectomy [6–13]. The preliminary results have revealed its superiority in aesthetics, postoperative pain, and convalescence [14, 15].

Among large series of reports for LESS adrenalectomy, APA is one of the most common surgical indications, comprising nearly 50% of all cases [11, 16–18]. However, LESS adrenalectomy is a challenging procedure which is commonly associated with poor ergonomics and instrument fighting and consequently with longer operative times when compared with conventional laparoscopic adrenalectomy [11, 17, 19]. In addition, the current evidence has not confirmed its comparable clinical effectiveness in treating APA. Thus, we conducted a study to compare the functional and clinical outcomes of LESS adrenalectomy with conventional multiport laparoscopic adrenalectomy in patients with APA.

## 2. Materials and Methods

### 2.1. Patients

We maintain a prospective database of all laparoscopic and LESS adrenalectomies performed in our department since 2007. This study is a retrospective review of prospectively collected data from this database and is approved by the ethics committee of Taipei Tzu Chi Hospital.

Between 2009 and 2014, 116 patients who had been clinically confirmed (by saline infusion and captopril tests) to have unilateral APA were included in this study. Those APA patients who had inconclusive abdominal contrast-enhanced CT results underwent adrenal scintigraphy with 131I-6 b-iodomethyl-19-norcholesterol (NP-59) SPECT/CT and/or adrenal venous sampling (AVS) for lateralization. Based on this database, we identified 45 APA patients who underwent LESS adrenalectomy (single-port group) and 71 who underwent conventional laparoscopic adrenalectomy (multiport group). The baseline characteristics of these 116 patients are listed in Table 1.

### 2.2. Operative Technique (Endoscopic Single-Port Adrenalectomy)

After induction of general anesthesia, the patient was placed in the prone jack-knife position. LESS adrenalectomy starts with a 2.0 to 3.0 cm skin incision just beneath the tip of the 12th rib. A homemade or commercial single-port was placed in position through the incision [13]. After pneumoretroperitoneum was established, an extended length 5 mm 30-degree laparoscope and conventional 5 mm laparoscopic instruments were used for subsequent manipulation. Step by step, the upper pole of the kidney was mobilized. Dissection of the adrenal gland started from its medial side. For the right adrenal tumors, the adrenal arteries, which cross the vena cava medially posteriorly,,were separated with a 5 mm LigaSure V Sealer/Divider (Covidien, Valleylab, Boulder, CO). Then, the short adrenal vein was located, controlled, and transected with LigaSure. Then, the right adrenal gland was completely mobilized by lateral and cranial dissection. For the left-sided adrenalectomy, dissection started from the inferior, medial border of the adrenal gland which is just above the anatomic landmark of the left renal artery. Then, the main left adrenal vein was identified, controlled, and transected with LigaSure. After that, an extended mobilization of the upper pole of the kidney was performed. Thereafter, the inferior part of the gland could be identified and dissected. Both adrenal glands with the surrounding fatty tissue were removed en bloc.

### 2.3. Operative Technique (Conventional Laparoscopic Adrenalectomy)

For multiport LPS adrenalectomy, the patient was placed in the flank position. Three to four trocars (10-5-5 mm or 10-5-5-5 mm) were used for the multiport procedure. The fourth 5 mm trocar was used for liver or spleen retraction over the subxiphoid incision. The 10 mm trocar was placed paraumbilically and the other 5 mm trocars were placed over subcostal incisions. After incision on the peritoneal reflection along the liver edge for right adrenal lesion or incision on the white line of Toldt for left adrenal lesion, the adrenal gland was identified. Once the adrenal vein was secured, the adrenal gland was mobilized from the upper pole of kidney with hook cautery or LigaSure V Sealer/Divider (Covidien, Valleylab, Boulder, CO).

### 2.4. Follow-Up

The patients returned regularly to the clinic for follow-up. Blood pressure and the levels of serum aldosterone, renin, and potassium were checked periodically. Postadrenalectomy clinical outcomes were judged on the basis of improvement in hypertension and hypokalemia. Hypertension was considered cured if blood pressure decreased to 140/90 mmHg or less shortly after adrenalectomy and antihypertensive medications were not required during the first year after adrenalectomy. Patients whose hypertension subsided within the first year but later returned were still classified as cured. Hypokalemia was considered resolved if serum potassium levels remained normal without potassium supplementation.

### 2.5. Statistical Analysis

Summaries of continuous variables were calculated as the mean ± standard deviation. Continuous variables were tested for normality with the Shapiro-Wilk's test. The Mann-Whitney *U* test and the independent samples *t*-test were used for continuous variables depending on the normality of the variable. A *p* value below to 0.05 was considered statistically significant. For categorized variables, the Fisher exact test and Chi-Square test were used. A *p* value below 0.05 was considered statistically significant. Statistical analyses were performed using SPSS for Windows (Version 18.0, PASW, SPSS, Inc., Chicago, IL). The learning curves were generated with curve fitting model with Microsoft Excel computer software.

## 3. Results

**Figure 1:**
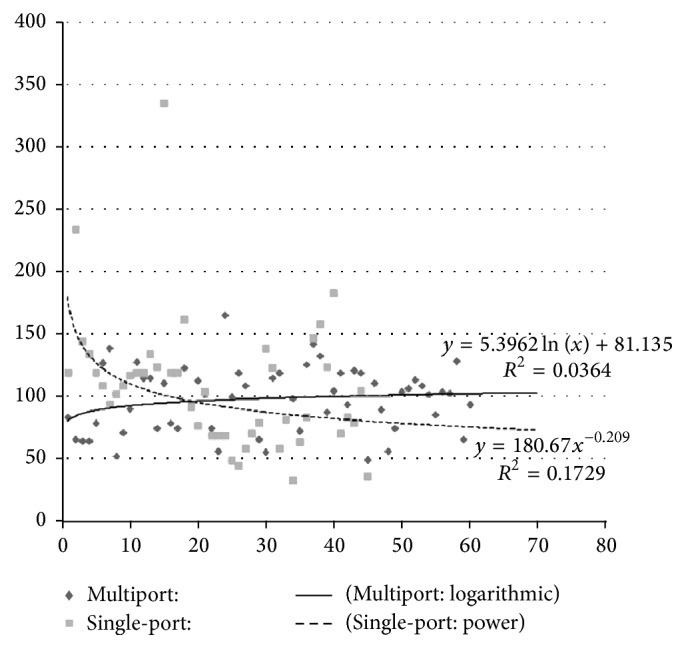
The fitting curves and the trend of operative times among patients of the 2 groups (*x*-axis represents the cumulative number of cases and *y*-axis represents the operative time (minutes)). There is a trend of shorter operative time among the later patients of the single-port group (the single-port curve best fits the power function), whereas the trend of operative time changes among the multiport group is more flat (the multiport curve best fits the logarithmic function).

The baseline demographic data is presented in Table 1. These two groups were matched in age, sex, BMI, operative time, tumor size, laterality of the tumor, preoperative aldosterone level, and mean time of follow-up. The only difference between groups is in the mean preoperative diastolic blood pressure. The single-port group had a higher mean diastolic pressure (98.4 ± 13.2 versus 88.8 ± 12.9 mmHg; *p* < 0.01) preoperatively, but this difference was not significant after surgery. In addition, there was a significantly lower mean postoperative systolic pressure in the single-port group (127.9 ± 9.7 versus 137.1 ± 15.5 mmHg; *p* < 0.01), but the difference was not significant before surgery. Histological examination confirmed cortical adenomas in all cases.  Figure 1 depicted the fitting curves and the trend of operative times among patients of the 2 groups. There is a trend of shorter operative time among the later patients of the single-port group (the single-port curve best fits the power function with *R* square of 0.173), whereas the trend of operative time changes among the multiport group is more flat (the multiport curve best fits the logarithmic function with *R* square of 0.036).

There was no mortality in the present series. There were no significant differences among groups in the incidence of surgical conversion and surgical complications (Table 2). There was one superobese female patient (BMI > 45) who converted to a conventional laparoscopic approach due to inability to maintain a reasonable retroperitoneal space during LESS approach and one open conversion in the multiport group due to major vessel and bowel injury. Neither of the groups had a port-site related complication (infection and/or incision hernia) during the period of follow-up (Table 2). The single-port group was associated with shorter hospital stay and the difference was significant (2.3 versus 4.0 days; *p* < 0.001). In our subgroup analysis of the operative time versus various demographic parameters among the single-port group in order to understand the factors affecting operative ergonomics, we found that those patients who had a BMI greater than 25 (123.2 ± 52.9 versus 89.9 ± 46.9 minutes; *p* = 0.01) were taller than 165 cm (131.9 ± 66.2 versus 92.5 ± 32.1 minutes; *p* = 0.02) or a right-sided tumor (128.0 ± 61.7 versus 92.7 ± 37.1 minutes; *p* = 0.03) were associated with a longer operation time when compared with their counterparts.

No difference in the incidence of improvement of blood pressure control (including cure and normotensive under medication) after surgery was observed between the single-port and multiport group (69% versus 62%; *p* = 0.05) (Table 3). In the subgroup analysis, postoperative cure of hypertension was achieved in 28/45 patients in the single-port group and 30/71 patients in the multiport group; the difference was significant (62% versus 42%; *p* = 0.036). Regarding the types of medication required for blood pressure control after surgery, no significant difference was observed between groups. Hypokalemia was resolved in all and no patient required potassium supplements after surgery.

## 4. Discussion

APA is one of the most common types of endocrine related secondary hypertension [1–3]. Unilateral laparoscopic adrenalectomy is the gold standard treatment for APA due to its high success rate, minimal morbidity, and short convalescence [4, 5]. LESS adrenalectomy is one of the most promising LESS procedures. Several preliminary and comparative studies have revealed its safety and feasibility in removing benign functional/nonfunctional adrenal tumors [11, 16–18, 20, 21]. Among these early series, APA is one of the most common indications for LESS adrenalectomy. However, functional outcome focusing on hypertension control after LESS adrenalectomy remains scarce in the literature [18, 21]. Besides, because the average size of APA was about 2-3 cm, APA is a good indication for LESS surgery. Therefore, a study comparing the hypertension control after LESS adrenalectomy and the current gold standard (laparoscopic adrenalectomy) is mandatory to confirm the treatment efficacy of LESS adrenalectomy in APA.

To the best of our knowledge, this is the first comparative study comparing the functional outcomes of APA after LESS and conventional laparoscopic adrenalectomy. In the current series, the improvement of hypertension after surgery (including cure and normotensive with medication) was comparable between groups. In our series, cure of hypertension was defined as blood pressure decreased to 140/90 mmHg (or below) after adrenalectomy and patients free from antihypertensive medication for the first year after treatment. The cure rates for APA after adrenalectomy have been reported in the literature, ranging from 33% to 87% [22]. When compared with open and laparoscopic counterparts with similar definitions of “cure” in the literature, the cure rate of the single-port group is comparable with those who have overall cure rates of 60% [22]. In addition, preoperative hypokalemia patients were all resolved in the early postoperative phase in both groups. Therefore, LESS adrenalectomy achieves similar hypertension control as conventional laparoscopic approach in APA patients is confirmed, based on our study.

There are only two meta-analyses that have compared LESS and conventional laparoscopic adrenalectomy for benign adrenal lesions thus far [14, 15]. These studies revealed comparable incidence of procedure related adverse events (complication and conversion) between the two techniques. The present cohort, which focused on APA patients undergoing adrenalectomy, also revealed a comparable minimal procedure related complication and low conversion rate when compared with the conventional laparoscopic counterpart.

According to our previously published study in comparing LESS and multiport adrenalectomy for benign adrenal tumors in 2012 [17], patients who underwent LESS adrenalectomy were associated with shorter convalescence when compared with those who underwent conventional laparoscopic approach due to less postoperative analgesic requirements. We propose that the LESS approach was done through the retroperitoneal route (possibly less violation of the bowel function) and thus early return of bowel function and oral intake. This phenomenon was also observed in the current trial. The single-port group was associated with a shorter hospital stay and the difference was significant (2.3 versus 4.0 days; *p* < 0.001). In contrast to our previous comparative trial that laparoendoscopic single-site adrenalectomy was associated with longer operating times, the current study revealed a comparable operative time between the two approaches (112.7 versus 97 minutes; *p* = 0.07). Although the current trial still enrolled cases of LESS adrenalectomy during the learning curve, the mean operative time decreased from the previous 154 minutes to the current 112 minutes after enrolling more cases after the curve. Therefore, based on our long-term observation, LESS adrenalectomy is associated with a better convalescence and possibly a similar surgical efficiency to conventional laparoscopic adrenalectomy with increased surgical experience.

LESS surgery is commonly associated with a single yet slightly larger port-site incision when compared with conventional laparoscopic surgery. Thus, concerns about port-site related complications might increase with LESS, especially among early case series performed with multiple fascial punctures in close proximity [23]. However, in our study, we did not find any case of port-site related complication in either group. In our practice, all the single-port incisions were achieved with a minilaparotomy technique which ranged from 2.5 to 3 cm, and then it was securely closed under direct vision at the end of each procedure. The other possible explanation for our lack of wound hernia complication is that all our LESS adrenalectomy cases were performed with the retroperitoneoscopic technique, and an intact peritoneal membrane might also help. Although the power of the current study was limited by its small sample size, after reviewing the recent studies that clearly address port-site complications with a larger sample size, increased risk of port-site related complications was not observed [23–26].

LESS adrenalectomy is associated with significant technical challenges when compared with conventional multiport laparoscopic adrenalectomy for the following reasons: loss of instrument triangulation, instrument clashing, especially with straight instruments, and the mind-set of the operator and assistant relying on their prior experiences of multiport laparoscopic surgery.  Figure 1 revealed that there is a trend of shorter operative time among the later patients of the single-port group. This implicates that through adapting to the unique surgical tricks of single-port surgery, we effectively overcame the learning curve of the ergonomics of the LESS. We have described our ergonomic tricks previously [27], and, by abiding to the following principles, novel traction/countertraction concept, one-instrument surgery most of the time (the other instrument was holding adjacent tissue away from the target of dissection and not completely seen under the monitor view), and employing special/longer laparoscopic instruments, and to keep one instrument static for tissue traction to provide enough space of movement for the other dynamic-moving instrument for dissection, the surgeon alternates the status of dynamic and static instruments flexibly to decrease the chances of instrument clashing and so forth. We successfully completed these 45 single-port adrenalectomies with the mean operative time numerically about 15 min longer than the mean of its multiport control, but the difference was not statistically significant (Table 1; *p* = 0.07).

Besides, we also identified that some demographic factors which might affect the instrument's torque (high BMI, increased abdominal, anterioposterior diameter, or body height) could make single-port more challenging. And the anatomical differences between left and right adrenal gland also affect the operation significantly, because, in a prone retroperitoneal approach, the right adrenal vein could not be approached early as in the left adrenalectomy. Thus, more delicate and time consuming procedure was necessary for LESS right adrenalectomy. All the above factors have been objectively confirmed in our subgroup analysis, those patients who had a BMI greater than 25 (123.2 ± 52.9 versus 89.9 ± 46.9 minutes; *p* = 0.01), a height more than 165 cm (131.9 ± 66.2 versus 92.5 ± 32.1 minutes; *p* = 0.02), or a right-sided tumor (128.0 ± 61.7 versus 92.7 ± 37.1 minutes; *p* = 0.03) were associated with a longer operation time when compared with their counterparts.

Although the current study had comparable baseline characteristics in both groups, the inherent limitation of the current trial is the patient selection bias due to nonrandomization. The surgeon bias might have enrolled more difficult and multimorbid patients to the multiport group, especially during the learning curve of single-port adrenalectomy. Thus, a prospective randomized trial after learning curve is mandatory to exclude this uncontrolled bias.

## 5. Conclusions

This is the first comparative study comparing the functional outcomes of APA after LESS and conventional laparoscopic adrenalectomy. Our experience confirmed that, in experienced hands, LESS adrenalectomy is a safe and feasible approach which was associated with comparable postoperative improvement of hypertension to the current gold standard treatment (laparoscopic adrenalectomy) in treating APA. In addition, LESS adrenalectomy for APA not only is associated with a shorter convalescence but also is an equivalent surgical efficiency when compared with conventional laparoscopic adrenalectomy. Therefore, LESS adrenalectomy for APA is a suitable alternative for surgeons experienced in the LESS approach.

## Figures and Tables

**Table 1:** Patient characteristics and surgical results.

	Single-port	Multiport	*p*
Number of patients	45	71	
Age (years)	50.8 (11.5)	51.3 (10.3)	0.82
Sex (M : F)	28/21	34/45	0.12
BMI	27.8 (7.2)	25.5 (4.4)	0.06
Operative time (minutes)	112.7 (52.3)	97 (25.8)	0.07
Tumor size (gm)	1.8 (0.5)	1.8 (0.7)	0.83
Right/left-side tumor	22/27	30/49	0.44
Aldosterone (ng/dL)	56.2 (20.7)	61.0 (44.2)	0.55
Systolic blood pressure (mmHg)			
Preoperative	152.6 (15.5)	152.7 (20.0)	0.96
Postoperative	127.9 (9.7)	137.1 (15.5)	<0.01
Diastolic blood pressure (mmHg)			
Preoperative	98.4 (13.2)	88.8 (12.9)	<0.01
Postoperative	82.7 (7.5)	84.2 (11.1)	0.36
Mean follow-up (month)	14.3 (12.2)	10.8 (3.8)	0.06

Data expressed as mean (SD) or absolute number of patients

BMI: body mass index.

**Table 2:** Surgical complications.

	Single-port	Multiport	*p*
Numbet of patients	45	71	
Conversion to			1.0
Laparoscopy	1	0	
Open	0	1	
Clavien grade/complication			0.27
Grade I	2	0	
Grade II	1	1	
Grade III	0	1	
Grade IV	0	0	
Port-site complication	0	0	
Hospital stay	2.3 (0.8)	4.0 (1.3)	<0.001

Data expressed as mean (SD) or absolute number of patients.

**Table 3:** Surgical outcomes of unilateral adrenalectomy in patients with aldosterone-producing adenoma.

	Single-port	Multiport	*p*
Number of patients	45	71	
*Blood pressure control after operation (%)*			0.056
Normotensive without medication (cure)	28 (62.2)	30 (42.3)	
Normotensive with medication	3 (6.6)	14 (19.7)	
Hypertensive	14 (31.2)	27 (38)	
*Medication after operation*			0.106
None	28 (62.2)	30 (42.3)	
Decreased	10 (22.2)	31 (43.6)	
No change	4 (8.8)	7 (9.8)	
Increased	3 (6.8)	3 (4.3)	
*Number of hypokalemic patients (%)*			
Before operation	30 (61.2)	30 (42.2)	
After operation (without supplementation)	0	0	

Data expressed as mean (SD) or absolute number of patients.
